# Barriers to management of opioid withdrawal in hospitals in England: a document analysis of hospital policies on the management of substance dependence

**DOI:** 10.1186/s12916-022-02351-y

**Published:** 2022-04-14

**Authors:** Magdalena Harris, Adam Holland, Dan Lewer, Michael Brown, Niamh Eastwood, Gary Sutton, Ben Sansom, Gabby Cruickshank, Molly Bradbury, Isabelle Guest, Jenny Scott

**Affiliations:** 1 Department of Public Health, Environments and Society, London School of Hygiene and Tropical Medicine, 15-17 Tavistock Place, London, WC1H 9SH UK; 2 Population Health Sciences, Bristol Medical School, University of Bristol, Oakfield Grove, Clifton, Bristol, BS8 2BN UK; 3 Department of Epidemiology and Public Health, University College London, 1-19 Torrington Place, London, WC1E 7HB UK; 4 Division of Infection, University College London Hospital, London, UK; 5 Department of Clinical Research, London School of Hygiene and Tropical Medicine, Keppel Street, London, WC1E 7HT UK; 6 Release, 61 Mansell Street, London, E1 8AN UK; 7 Bristol Medical School, University of Bristol, Oakfield Grove, Clifton, Bristol, BS8 2BN UK; 8 Severn Foundation School, Park House, 1200 Parkway, Bristol, BS34 8YU UK; 9 Southmead Hospital, Southmead Road, Westbury-on-Trym, Bristol, BS10 5NB UK; 10 Department of Pharmacy & Pharmacology, University of Bath, Claverton Down, Bath, BA2 7AY UK

**Keywords:** Opioid substitution therapy, Opioid dependence, Opioid withdrawal, Hospital policy, People who use drugs, Discharge against medical advice, Stigma, Opioid overdose, Community-based participatory research, Document analysis

## Abstract

**Background:**

People who use illicit opioids are more likely to be admitted to hospital than people of the same age in the general population. Many admissions end in discharge against medical advice, which is associated with readmission and all-cause mortality. Opioid withdrawal contributes to premature discharge. We sought to understand the barriers to timely provision of opioid substitution therapy (OST), which helps to prevent opioid withdrawal, in acute hospitals in England.

**Methods:**

We requested policies on substance dependence management from 135 National Health Service trusts, which manage acute hospitals in England, and conducted a document content analysis. Additionally, we reviewed an Omitted and Delayed Medicines Tool (ODMT), one resource used to inform critical medicine categorisation in England. We worked closely with people with lived experience of OST and/or illicit opioid use, informed by principles of community-based participatory research.

**Results:**

Eighty-six (64%) trusts provided 101 relevant policies. An additional 44 (33%) responded but could not provide relevant policies, and five (4%) did not send a definitive response. Policies illustrate procedural barriers to OST provision, including inconsistent application of national guidelines across trusts. Continuing community OST prescriptions for people admitted in the evening, night-time, or weekend was often precluded by requirements to confirm doses with organisations that were closed during these hours. 42/101 trusts (42%) required or recommended a urine drug test positive for OST medications or opioids prior to OST prescription. The language used in many policies was stigmatising and characterised people who use drugs as untrustworthy. OST was not specifically mentioned in the reviewed ODMT, with ‘drugs used in substance dependence’ collectively categorised as posing low risk if delayed and moderate risk if omitted.

**Conclusions:**

Many hospitals in England have policies that likely prevent timely and effective OST. This was underpinned by the ‘low-risk’ categorisation of OST delay in the ODMT. Delays to continuity of OST between community and hospital settings may contribute to inpatient opioid withdrawal and increase the risk of discharge against medical advice. Acute hospitals in England require standardised best practice policies that account for the needs of this patient group.

**Supplementary Information:**

The online version contains supplementary material available at 10.1186/s12916-022-02351-y.

## Background

The United Kingdom (UK) has the largest reported population of people who use non-prescribed opioids in Europe. Latest available English surveillance data report over 250,000 people using heroin or other illicit opioids in 2016/17 [1], with 140,294 in treatment for opioid dependence in 2020/21 [2]. The rate of hospital admission in this population is several times greater than people of the same age in the general population [3], with most admissions relating to long-term health conditions, injuries and bacterial infections [3–7]. Delays to seeking treatment are common, most admissions are unplanned [4, 8, 9], and many result in discharge against medical advice [10–15]. Patients who leave hospital prematurely are more likely to be readmitted and have higher all-cause mortality [11–14]. Hospital discharge is a particularly risky time for people who use heroin as they may leave hospital in an unfamiliar neighbourhood and use drugs in public places, often while unwell and with reduced opioid tolerance, which are all risk factors for overdose. A recent study in England demonstrated one in fourteen fatal opioid overdoses happen shortly after hospital discharge and the risk in this period is four times higher than usual [16].

Qualitative research with people who inject drugs in London, UK, has found that fear of opioid withdrawal is a primary barrier to timely hospital presentation and completion of inpatient care for injecting-related infections [9]. This is corroborated by other studies demonstrating that opioid withdrawal in hospital can cause severe distress, with patients often self-discharging to collect community opioid prescriptions or use illicit opioids [17, 18]. Other factors contributing to late presentation and discharge against medical advice include inadequate pain management, competing priorities precluding hospital attendance and treatment completion, and stigmatising attitudes toward people who use illicit drugs among hospital staff [9, 17–20].

In the UK, opioid substitution therapy (OST)—the prescription of methadone or buprenorphine—is recommended for opioid dependence [21]. This is well evidenced to reduce drug-related health harms including HIV [22], hepatitis C [23], and mortality [24]. Continuity of OST provision between community and inpatient settings is crucial to prevent opioid withdrawal, reduce discharge against medical advice and facilitate effective treatment of presenting medical issues [10, 25, 26]. National guidance recommends the development of local pathways to ensure the prompt prescription of OST in hospital [27]. Access may, however, be limited by concerns about the safety of OST, particularly in combination with illicit drugs and other medications when the patient’s tolerance may be unknown [27]. Hospitals are directed to identify a list of critical medicines, for which administration should not be omitted or delayed. Until recently, this categorisation has been supported by the 2010 Specialist Pharmacy Service Omitted and Delayed Medicines Tool (ODMT)  [28]. The ODMT categorised risk depending on the potential consequences for patients not receiving medications. We sought to understand if and how hospital policies contributed to OST delay by first, assessing OST categorisation in the few publicly available hospital critical medicines lists and in the 2010 ODMT, and second, requesting and reviewing policies on substance dependence management from hospital trusts, the organisational unit of England’s National Health Service (NHS). An initial outcome of this work was to inform the reclassification of drugs for substance dependence management in the ODMT. This collaborative process with the Specialist Pharmacy Service contributed towards a more comprehensive review of the ODMT in 2020 and its subsequent discontinuation to make way for a more holistic, patient-centred and context-specific framework.

## Methods

### Approach

Our approach was informed by principles of community-based participatory research, emphasising equitable collaboration between academics and community partners to integrate knowledge and action to reduce health disparities [29]. Research was conducted by a multidisciplinary team of academics in collaboration with the drug policy charity Release, clinicians, people with lived experience of opioid dependence, medication safety officers, pharmacists and public health policy makers. It was conducted in response to findings from the ‘Care and Prevent’ study, which aimed to improve care for skin and soft tissue infections among people who inject drugs in the UK [4, 9, 30–36]. We undertook a document analysis [37] of hospital policies, utilising principles of content analysis [38] and reported the study with reference to the Standards for Reporting Qualitative Research [39].

### Sampling

We identified trusts in England from the NHS website [40]. Trusts were included if they acutely admit patients to treat physical health problems. We excluded ambulance, children’s, community and mental health trusts, and tertiary centres offering only planned admissions. Whether inclusion and exclusion criteria were met was discerned from publicly available information describing services provided and/or responses to our requests.

### Data collection

We contacted trusts between October 2019 and January 2020 requesting local policies pertaining to substance dependence management, under the terms of the Freedom of Information Act 2000 [41]. In 2021 (April to July), we sent follow-up requests to trusts who had not provided relevant policies, asking for policies which were in place in January 2020 to allow comparability with trusts who replied to the earlier request. The lengthy period between the two data collection points was informed by sensitivities to trust workloads during the COVID-19 pandemic. We received policies with different focuses, including substance dependence management; pain and peri-operative management for people who use drugs; and drug use in pregnancy. We included any policies that were in use in January 2020 containing practical guidance on the management of opioid withdrawal and/or OST provision.

### Analysis

Document analysis [37] of hospital policies took place in three stages. First, we identified key areas of interest using policies obtained from the first request in close collaboration with people with lived experience of opioid dependency (see PPI section below). Second, we developed a preliminary data extraction tool to collate guidance from these policies related to key areas of interest to gain familiarity with the data. Third, we revised the data extraction tool to undertake a content analysis [38] of the final sample of policies. We categorised policy characteristics and content under twelve headings, each with 2–16 sub-headings. Headings included OST continuation, OST initiation, drug testing, discharge planning and communications with community drug services. Sub-headings referred to specific elements of guidance related to the headings, for example, under OST continuation: recommendations on confirming community OST prescriptions and advice if this could not be confirmed out of hours. ‘Other’ sub-headings were included to capture extraneous information. Guidance from the policies were categorised by AH, MBrad and IG who regularly communicated to discuss approach. We extracted verbatim quotes (for example, to illustrate typical language). Analytic memos were recorded and discussed between AH, MH and JS. Summary statistics were produced demonstrating category prevalence. We present results under five headings, chosen to highlight policy discrepancies and barriers to timely OST.

### Patient and public involvement

Our community-based participatory approach predicates community involvement, including people who have received OST and/or used illicit opioids. Three workshops were held in 2019 with 26 people who use drugs in London to discuss Care and Prevent study findings, with a focus on understanding barriers to hospital care. Participants emphasised OST delay as a key factor. Release, staffed by people with legal, medical, and lived drug use expertise, initially led the policy request. Release staff, including those with lived experience of opioid use, reviewed a subset of policies and reported key themes at a meeting attended by drug treatment service providers, clinicians, public health policy advisors, pharmacists, and people with lived experience of OST and/or opioid dependency from throughout the UK. Participants reached a consensus view that current hospital policies were suboptimal and contributed to risk. Informed by this community consultation, we presented the case for a review of the ODMT to the Specialist Pharmacy Service and, in collaboration with Release, conducted the formal document analysis presented here.

## Results

### OST risk categorisation

**Fig. 1:**
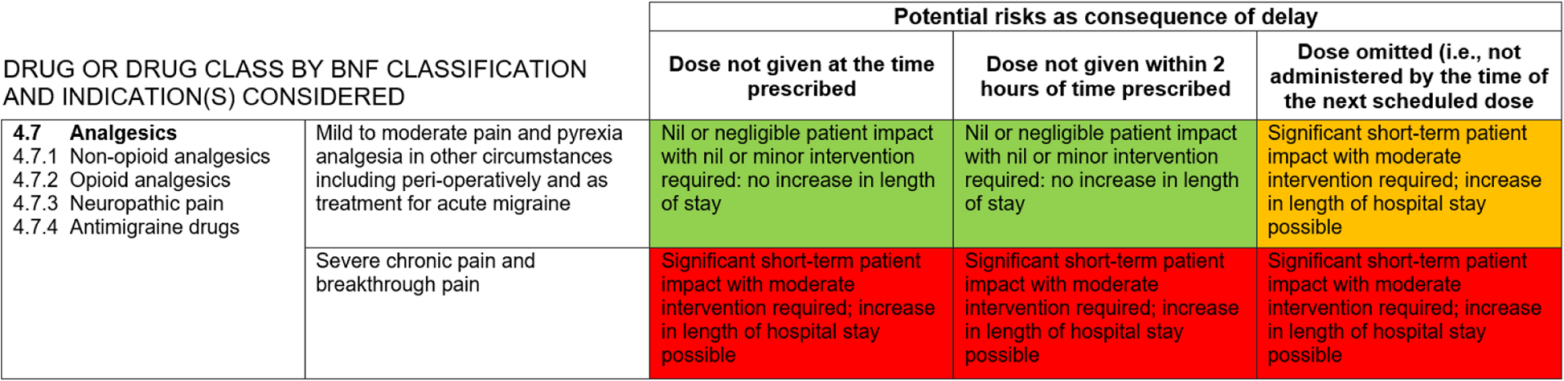
The categorisation of analgesics, including opioid analgesics, in the 2010 Omitted and Delayed Medicines Tool, produced by the Specialist Pharmacy Service to inform the NHS critical medicine categorisation. Here, opioid analgesics for severe chronic pain and breakthrough pain are categorised as ‘high risk’ if delayed or omitted

**Fig. 2:**
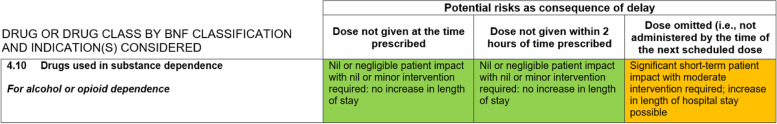
The categorisation of drugs used in substance dependence in the 2010 Omitted and Delayed Medicines Tool. This comprises one category, combining drugs used for alcohol or opioid dependence. Unlike opioid analgesics for severe pain (Fig. 1), drugs used for opioid dependence are here classed as low risk if delayed and medium risk if omitted.

Our exploratory assessment of hospital critical medicine lists illustrated inconsistencies in OST classification. Of the five available through a public website search, one did not include any medicines for substance dependence or analgesic opioids, one included analgesic opioids only, two specifically included methadone and buprenorphine and one listed ‘drugs for opioid dependence’ as critical in regard to omission but not delay. To understand this inconsistency, we reviewed the ODMT which, at that time, informed local critical medicines lists. Medications were deemed critical if classed as high risk (red) for either delay (‘dose not given at the time prescribed’ OR ‘dose not given within 2 h of the time prescribed’) or omission (‘not administered by the time of the next scheduled dose’). Only drugs for alcohol or opioid dependence were considered under ‘Drugs used in substance dependence’. Unlike analgesics for ‘severe chronic pain and breakthrough pain’ (Fig. 1) drugs for substance dependence were categorised as low risk if delayed and medium risk if omitted (Fig. 2).

### Trust responses and policy characteristics

**Fig. 3:**
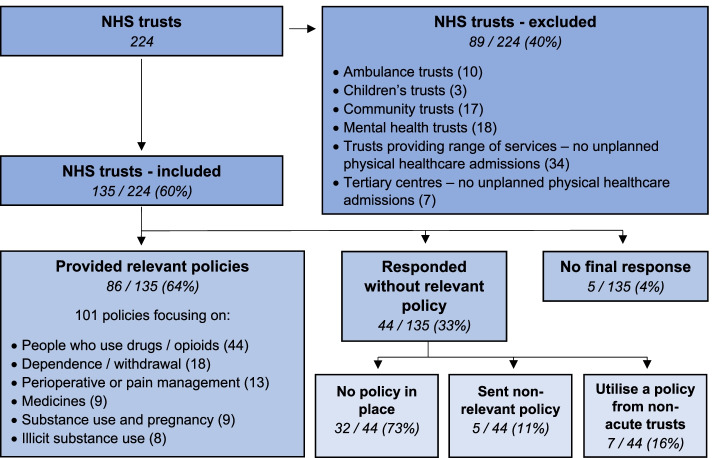
NHS trust policy inclusion flowchart

We identified 224 NHS trusts in England in January 2020, of which 135 met our inclusion criteria. Of these 135 trusts, 86 (64%) provided one or more relevant policies (including duplicates from two pairs of trusts with joint policies), 44 (33%) responded without a relevant policy and five (4%) did not send a definitive response, including to follow-up requests (Fig. 3). Seven trusts stated they used policies provided by local mental health trusts, but these were not provided.

Our analysis includes 101 polices from 86 trusts. Of the 86 included trusts, 35 (14%) only provided policies related to pain, peri-operative management or substance use in pregnancy (with sections on withdrawal) and five (6%) only provided general medicine policies with sub-sections on OST.

In most cases, authors of policies were listed, usually including a pharmacist and sometimes clinicians from community or liaison drug treatment services, anaesthetists, acute and emergency medicine physicians, psychiatrists, and midwives. Of the 101 relevant policies, 32 (32%) stated community drug treatment services were consulted in their development. None indicated patient consultation. Twenty-seven (28%) policies were overdue review, and 14 (14%) did not include enough information to ascertain whether this was the case.

### Policy review findings

Below, we present findings under five headings. The first three are processes including barriers to timely OST. The latter two highlight general procedural barriers to OST prescription and issues of policy comprehensiveness, clarity, and tone. We forefront sections with relevant national guidance to contextualise findings.

#### Continuation of community prescriptions

*UK Guidelines on Clinical Management for Drug Misuse and Dependence* specify when people on community OST are admitted to hospital, OST prescription information is required as ‘a matter of high priority’. Prior to prescribing, an assessment is required: ‘where possible ascertaining independently when the last prescribed dose of OST was dispensed and, if possible, when it was consumed’ [27].

All policies reviewed stressed the need to independently confirm patients’ doses of community OST prior to prescribing. The means of verification was not always specified, but usually included contacting the prescriber (drug treatment service or general practitioner) and/or the dispenser (community pharmacist), with some policies requiring confirmation of when doses were last collected and if consumption was supervised. Of the 86 trusts, 18 (21%) provided alternative verification options such as contacting key workers or drug liaison services, online databases, previous hospital notes, drug charts or discharge summaries. Three trusts required written confirmation of doses from the prescriber or dispenser. Only one policy included an option of patient verification, while some explicitly stated that patients are not a trustworthy source for dose confirmation.

Verifying OST doses was often restricted to pharmacy or community drug treatment opening hours. Of the 86 trusts, 17 (20%) provided no guidance for cases when verification was not possible, and six (7%) explicitly stated that no OST should be prescribed in these circumstances. Regardless of whether community prescriptions were confirmed, some policies advised gradually increasing (titrating) doses ‘when in doubt’ or stated a lower initial dose was ‘still reasonable’. Thirty-three (38%) trusts recommended re-titration if a certain period of time had elapsed since the previous dose (normally 72 h, with a range of 48–120), often with the same regimens used for OST-naïve patients, and six recommended re-titration if consumption in the community was not supervised or if recent doses had been missed. Most guidance pertained to methadone, with only 37 (43%) trusts providing guidance on continuation of buprenorphine prescriptions. Notably, buprenorphine was not mentioned at all by 19 (22%) trusts.

#### New or unconfirmed prescriptions

National guidance discusses OST initiation in hospital with the proviso that ‘for patients not on OST, or where there is uncertainty about recent compliance, it is appropriate to exercise particular care’ [27]. This entails prescribing methadone in small divided doses, and titrating against opioid withdrawal symptoms, with an initial dose of no more than 10mg four times a day. The reviewed policies provided inconsistent guidance on how to manage withdrawal for patients who were OST-naïve or whose community prescription could not be confirmed.

Of the 86 trusts, 67 (78%) described a regimen for OST initiation in hospital. Others suggested this was possible but included no practical information on how to do so, sometimes advising referral to a specific hospital team or community drug service. Some did not mention that OST initiation was an option, and five (6%) prohibited it. Some trusts advised that non-opioid medications, such as anti-emetics, should be prescribed in lieu of or before OST for symptomatic relief and a few advised treating opioid withdrawal with oral morphine, codeine phosphate, or dihydrocodeine instead of methadone or buprenorphine.

Methadone regimens recommended for new or unconfirmed prescriptions included regular small (*statim*) doses, as required (*pro re nata*) regimes or various induction regimens to titrate to a regular dose. Twelve (14%) trusts stated no more than 30mg of methadone should be administered on day one (less than national guidance), and two stated no more than 20mg. Some policies did not allow for methadone doses to subsequently increase, and others limited dose increments to 5–10mg over 72 h. Only 23 (27%) trusts provided the option of initiating buprenorphine. Guidance for buprenorphine titration was not always provided; where stated, this was inconsistent between trusts, with some requiring specialist team input, which was not required for methadone initiation.

Fifty-five (64%) trusts required clinical symptoms of opioid withdrawal such as tachycardia, sweating or tremor before OST could be initiated, with some requiring specific scores on the Clinical Opiate Withdrawal Scale. Only five (6%) policies stated OST could be initiated for subjective symptoms, and most policies did not mention the psychological impact of withdrawal, which patients might face before clinical symptoms are observed. Many policies stated that patients do not develop withdrawal symptoms from methadone for 24 h and advised against prescribing any OST unless patients were admitted, sometimes for at least 24 and in one case, 48 h.

#### Discharge

Hospital discharge can interrupt OST if community services are not notified in advance. This is a particular risk if patients are discharged late on Friday or during the weekend. National guidelines highlight admission to hospital as an opportunity to engage people with community drug treatment services, emphasising the need to ensure that community prescriptions are restarted and prescriptions commenced in hospital are continued on discharge [27]. Despite this, 11 (13%) trusts did not include information on restarting community prescriptions post-discharge, and 26 (27%) of the 78 trusts that indicated OST could be initiated in hospital did not include information on facilitating continuation of new OST prescriptions on discharge. One policy explicitly stated hospital clinicians should not arrange continuation of new prescriptions requiring patients to self-refer to community services.

Fifty-seven (66%) trusts advised the hospital pharmacy could provide OST doses for a patient to take away in some circumstances. This often needed to be agreed with community drug services, which may not be possible if discharge occurred out of service hours. The remaining 29 (34%) trusts either did not mention providing OST to take away or explicitly stated it should not be. Most policies recommended community drug treatment services should be notified when their clients, or other patients with drug dependency were admitted to hospital and discharged; however, this was not always the case and advice was not always prescriptive.

#### Procedural barriers

Procedural barriers included drug test requirements and specifications for particular staff, combinations of staff or specialist teams to assess patients, confirm community doses and/or write OST prescriptions. Thirty-two (37%) trusts had policies referring to drug liaison teams in their hospitals. While liaison teams offer many benefits, these were potentially undermined by protocols requiring their presence for OST prescription and withdrawal management, posing barriers if teams were poorly resourced or operated in limited hours.

Urine drug testing is mentioned in national guidance as one form of verification prior to continuation of community OST prescriptions in hospital [27]. This is however, provided as an option rather than mandatory. Of the 86 trusts, 62 (72%) recommended drug tests for opioid dependent patients, at least in some situations. These references mainly referred to urine drug screens (UDS) and in some cases oral swabs. Some trusts required a laboratory test result positive for opioid substitution medications or opioids before OST could be prescribed rather than a point-of-care test. Of the 86 trusts, 14 (16%) required a positive UDS prior to OST prescription, regardless of whether a community prescription was confirmed; 13 (15%) required a positive test for unconfirmed or new prescriptions; and 15 (17%) suggested it was preferable to have a positive test prior to prescription. Of the 42 trusts which required or recommended positive UDS prior to OST prescription, only 10 (24%) stated point of care tests were potentially available and 19 (45%) required laboratory testing. In some cases, policies highlighted local laboratories could not process UDS out of office hours, so samples needed to be couriered to other hospitals. One trust, which required a positive UDS prior to initiating OST stated it could take up to 2 weeks to receive test results (it was not clear if point of care tests were also available). Few policies highlighted limitations of UDS; only five mentioned the possibility of false negative results, and many did not give the time frames following drug use in which test results would be the most accurate.

#### Comprehensiveness, clarity, and tone

Policy comprehensiveness and length varied considerably, ranging from 1 to 72 pages. Of the 86 trusts, 55 (64%) provided care pathway flow charts; however, these varied in detail and clarity. Some of the language used was unclear, for example, referring to ‘detoxification’ when the guidance related to maintenance therapy. Some policies specifically highlighted detoxification was not recommended in an acute setting because of the increased risk of overdose following discharge; however, many recommended measures which would lead to reduced tolerance by withholding OST.

Statements such as ‘opioid withdrawal is not a life-threatening condition, but opioid toxicity is’ were common. Most policies framed considerations about OST in terms of safety. These, however, often focused on verifying patients’ usual OST dose to prevent overdoses, as opposed to other considerations such as potential interactions between medicines, the possibility of increased opioid tolerance related to heroin use alongside OST in the community, or preventing premature discharges. Of the 86 trusts, 32 (37%) provided no information on contraindications and OST drug interactions. Only 42 (56%) provided policies that referred to opioid overdose management.

Many policies advised measures which appeared mistrustful of patients. Of the 86 trusts, 31 (36%) recommended OST consumption was supervised, with some instructing patients should be made to speak or swallow water to prove they were not holding medication in their mouth. Some recommended regular drug tests to monitor for illicit drug use, with one maternity guideline stating new mothers must be informed that if a test were positive, they may be discharged while their baby remains in hospital until fit for discharge. Of the 62 trusts that recommended UDS, 46 (74%) did not state a need to obtain patient consent prior to the test and six (10%) advised observing the patient urinate. Some policies advised restricting visitors, and specified patients should not be allowed to leave the ward. Four (5%) trusts recommended asking patients to sign behavioural contracts; two of which explicitly stated that medical treatment could be withdrawn if patients did not abide by contract terms.

The language used to describe patients often included potentially stigmatising terms such as ‘user’, ‘misuser’, ‘abuser’ and ‘addict’. Some policies discussed ‘sanctions’ and maintaining ‘a degree of suspicion’. Five (6%) trusts stated OST should only be initiated if patients had stopped or were motivated to change some aspects of their drug use. In some cases, policies explicitly recommended care differing from that for the general patient population, for example: ‘Patients with a history of drug abuse often have unreasonably high expectations. Alleviation of all pain is not a goal’.

## Discussion

‘Well thought-out protocols and guidance for how hospital staff can respond to people who may have problems from their use of drugs or alcohol, which address the full pathway from before admission to the point of discharge, will support better outcomes for the patient and clinicians and can reduce the likelihood of re-presentation’ [27].

We found that hospital policies relating to opioid dependence varied widely and often precluded timely OST. Many policies did not follow national guidance. Of the 135 NHS trusts who replied to our requests, 37 (27%) indicated that as of January 2020, they did not have a local policy in place informing the management of opioid withdrawal and/or OST prescription. People who receive community OST prescriptions and/or are dependent on illicit opioids are a marginalised population facing multiple barriers to accessing healthcare. This is critical to address given high and rising rates of morbidity and mortality among people who use drugs and the individual suffering this represents. Recognised is the role of stigma in dissuading people who use drugs from accessing care, with multiple studies illustrating discriminatory attitudes and practices toward this group in medical settings [20, 42, 43]. Others have identified how institutional policies may perpetuate or challenge stigma and discriminatory practice [44, 45]. Our study, however, is the first to comprehensively review a national census of hospital substance dependence policies, crucial for understanding and transforming healthcare inequities and outcomes for the most marginalised.

**Fig. 4:**
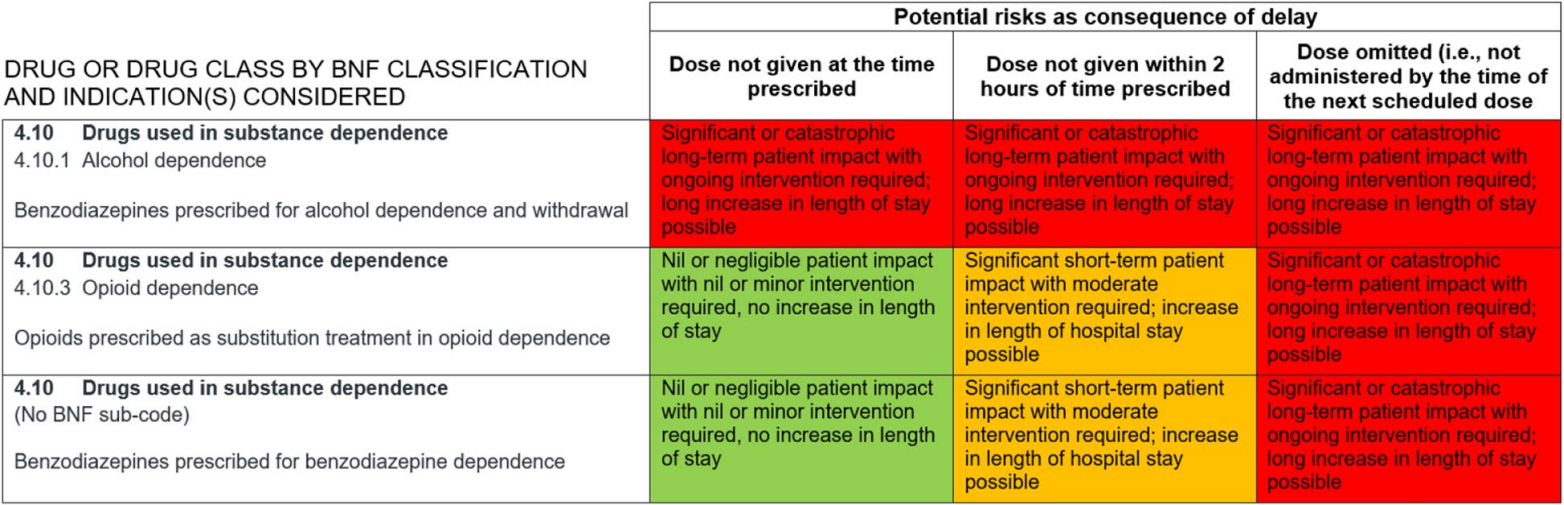
The 2020 revised version of drugs used in substance dependence in the Omitted and Delayed Medicines Tool. Following engagement with the Specialist Pharmacy Service, and consultation with Public Health England, community stakeholders and clinicians, a distinction was introduced between drugs used in alcohol, opioid and benzodiazepine dependencies with the risk categorisation increased for all. The ODMT is now undergoing substantive revision, with the Specialist Pharmacy Service concerned to produce a framework which reflects the complexity of medicine safety decisions, with attention to context and patient diversity

While NHS trusts are responsible for developing local policies, these are underpinned by reference to national guidelines and toolkits. The responsiveness of the Specialist Pharmacy Service to considering our research findings, then collaborating to review and reframe the categorisation of drugs for substance dependence, illustrates the potential for change more broadly. We made the case that the 2010 ODMT classification of drugs for substance dependence was problematic for two reasons. First, it combined two substance dependencies (alcohol and opioids) with distinct risk profiles into one classification and negated consideration of other dependencies (e.g., benzodiazepines). Second, the risk categorisation (low for delay, medium for omission) did not reflect risks associated with psychological distress (including through fear of withdrawal) and concomitant use of illicit drugs on wards, self-discharge and treatment interruption. In consultation with community stakeholders, clinicians and Public Health England, the Specialist Pharmacy Service worked with us to reconfigure the categorisation of drugs used in substance dependence. From one category (drugs used in alcohol and opioid dependence), three were demarcated (alcohol, opioid, benzodiazepine) with risk severity increased for all (Fig. 4). This process proved to be an interim step in a broader reconsideration of the role of the ODMT by the Specialist Pharmacy Service. It is to be withdrawn, with a view to developing a more context-specific and holistic framework to inform frontline care.

This iterative process of stakeholder consultation and resource refinement indicates an openness by organisations, such as the Specialist Pharmacy Service, to support broader and more substantive revisions to local NHS policies for substance dependence. The reconfiguration, and now potential discontinuation of the ODMT in its current form, paves the way for further changes to improve care for people who use drugs more broadly. The prior designation of medication delay for opioid dependency as no or low risk is likely to have underpinned or reinforced statements common to hospital policies such as ‘opioid withdrawal is not a life-threatening condition, but opioid toxicity is’. This statement emphasises the proximal risk of opioid overdose (toxicity), arguably well managed within a hospital setting, while negating consideration of the broader ways in which opioid withdrawal may constitute a life-threatening situation. This is particularly the case when withdrawal leads to patients self-discharging and using illicit opioids outside of the hospital setting with reduced opioid tolerance related to inadequate OST provision. A recent study showed that people who use illicit opioids are four times more likely to fatally overdose soon after hospital discharge than usual, suggesting a need for better continuity of care [16].

Findings illustrate policy and procedural barriers to OST, including inconsistent application of national guidelines across trusts. Policies highlighted the need for patients to abstain from illicit drugs when in hospital but many recommended OST regimens which would be unlikely to facilitate this without withdrawal symptoms, as opposed to in the community, where OST may be titrated alongside continued heroin use, and more rapidly than some hospital policies recommend. The primary role of OST initiation in hospital is to prevent opioid withdrawal in hospital, regardless of whether it is continued following discharge. We note with concern policies that require patients to demonstrate motivation ‘to change at least some aspects of their drug misuse’ prior to OST initiation. Requirements such as behavioural contracts and for urine sample provision and OST consumption to be observed would not be required for other patient groups receiving opioids as analgesia; illustrating how stigma towards people who use drugs can be structurally embedded within institutional protocols. Broader-based stigma reduction interventions focusing on attitudinal change in hospital settings [46–48] can only succeed if underpinned by non-pejorative patient-centred policies.

While we have focused on reporting policy barriers to timely OST, guidance with the potential to improve the care of people with opioid dependence and facilitate prompt OST prescription were also identified (see Supplementary file). These, with findings reported in this paper, will inform the development of a standardised policy template for opioid dependence management to be evaluated by our team as part of a broader intervention to improve care for people with opioid dependence in NHS hospital trusts^1^. Vitally, ongoing work to improve the care of people who use drugs should draw on the lived expertise of this patient group; none of the policies we reviewed indicated patients had been involved in their development.

### Limitations

We cannot be confident all relevant policies were identified. The detail of responses varied considerably, and some trusts indicated they used policies from non-acute trusts that were not provided. We analysed the policies on face-value; however, there may be differences between policy and practice if clinicians were unaware of or chose to ignore policies, providing care either better or worse than recommended. Additional procedural barriers may exist not described in policies, for example pharmacy procedures governing medication availability and availability of point of care tests.

## Conclusion

We focus on highlighting policy barriers to timely management of opioid withdrawal in acute hospital settings with the aim of transforming and standardising NHS practice. Our review of 101 policies from 86 acute hospital NHS trusts demonstrates variability in approach, including deviation from national guidelines. Policy barriers to timely OST in hospital pose a risk to the safety of patients with opioid dependency. Although opioid withdrawal is commonly framed as ‘low risk’ in relation to opioid overdose, we contend that these events are inextricably linked and as such should be accorded equal priority.

**Table:** 

**Summary of research** *What is already known on this topic* • Delayed presentation, discharge against medical advice and hospital readmissions are common amongst people who are dependent on non-prescribed opioids such as heroin. • Qualitative studies demonstrate that a key factor driving this is fear of opioid withdrawal in hospital, as well as inadequate pain management, restrictions on movement and visitors, and stigmatising attitudes among healthcare providers. • National guidelines recommend NHS trusts develop local pathways to ensure the prompt and effective prescription of OST in hospital, but the coverage and content of these policies has not been explored. *What this study adds* • A quarter of hospitals could not provide a local policy governing opioid withdrawal and OST prescription as of January 2020. • Policies include highly variable OST procedures, inconsistencies with national guidelines, and barriers to timely opioid withdrawal management for hospital inpatients. • A national policy template developed with patient involvement would be beneficial to ensure that the management of substance dependence in NHS acute hospitals is equitable, optimised and patient centred.	

## Supplementary Information

**Additional file 1:.** Examples of alternative practice.

## Data Availability

Trust level data are available from the corresponding author (magdalena.harris@lshtm.ac.uk). Trust consent for data sharing was not obtained; however, policies could otherwise be obtained by members of the public with Freedom of Information requests.

## Abbreviations

COVID-19: Coronavirus disease 2019
HIV: Human immunodeficiency virus
NHS: National Health Service
OST: Opioid substitution therapy
ODMT: Omitted and Delayed Medicines Tool
UDS: Urine drug screen
UK: United Kingdom

## Footnotes

1 Improving Hospital Opioid Substitution Therapy (iHOST): evaluation of an intervention to reduce late presentations, discharge against medical advice and repeat admissions among people who use opioids. Funded by National Institute for Health Research [NIHR133022].
