# Supplementary material for: Barriers to management of opioid withdrawal in hospitals in England: a document analysis of hospital policies on the management of substance dependence
Source: BMC Med. 2022 Apr 14;20:151. doi: 10.1186/s12916-022-02351-y (PMC9007696; doi:10.1186/s12916-022-02351-y)
Supplement: Supplementary file 1 — Additional file 1:. Examples of alternative practice. [file 12916_2022_2351_MOESM1_ESM.docx]

**Appendix A: Examples of alternative practice**

***Medicine reconciliation and continuation of community prescriptions***

Bradford Teaching Hospitals NHS Foundation Trust allowed for patients to act as a source of OST dosage confirmation if it is impossible to independently verify this out of hours but emphasise questioning the “veracity” of patient information provided. A few policies allowed for patient medication bottles as a valid source, provided there is no evidence the labels have been tampered with.

Some patients continue to use street heroin ‘on top’ of their OST prescriptions in the community, meaning they will suffer from withdrawal symptoms even when receiving their prescribed OST dose. York Teaching Hospitals NHS Foundation Trust explicitly highlighted this and indicated that as required OST doses may be necessary on top of regular doses.

One in five policies gave options for alternatives to normal OST medications if patients are nil by mouth for surgical procedures or certain investigations.

Although few policies mentioned the possibility that patients could be prescribed injectable OST for self-administration in the community, Cambridge University Hospitals NHS Foundation Trust recommended patients should be allowed to continue self-administering if clinically appropriate and discussed with the community drug treatment service.

***New and unconfirmed doses***

Some trusts recognised that when patients are under closer observation in hospital than in the community it may be possible to titrate doses of OST more rapidly than proposed by national guidance. One in five trusts recommended that more than 40mg of methadone could be administered in the first day of treatment, with one trust recommending up to 70mg in split doses.

Five trusts recommended that OST could be initiated to treat subjective symptoms of withdrawal, prior to waiting for the development of clinical symptoms.

***Discharges***

A minority of trusts provided discharge checklists, or proformas to send to community drug services, providing an opportunity to ensure appropriate information about changes to OST prescriptions is shared, and potentially serving as an opportunity to prompt clinicians to discuss, for example, harm reduction advice, blood borne virus screening, and to ensure the provision of naloxone.

***Procedural barriers***

St George’s University Hospitals NHS Foundation Trust explicitly stated that because of its limitations and the risk of false negatives UDS are not clinically helpful and should not be performed. Half of the trusts did not require or recommend UDS prior to OST prescription.

***Clarity, comprehensiveness, and tone***

In some cases, comprehensive and useful guidance was provided on drug interactions, overdose management and other safety concerns. For example, Lewisham and Greenwich NHS Trust, for example, provided specific detailed guidance on the investigation and management of QT prolongation during methadone induction.

Many trusts took advantage of the opportunity to provide guidance on wider issues which may complicate the care of people who are dependent on drugs. This included guidance on prescribing analgesia or gaining venous access; the need to consider contemporaneous withdrawal from other drugs; referring patients to housing or other support services; offering screening for blood borne viruses; providing naloxone; and offering harm reduction advice.

Some trusts highlighted the need to challenge negative preconceptions about people who use drugs, for example Lancashire Teaching Hospitals NHS Foundation Trust, whose policy stated:

*‘Drug users cross all social boundaries, and many users continue in full time employment. Drug users are often aware that some people have a negative, judgemental opinion of them and are consequently concerned that they will be treated unfairly by health care providers.’*
